# Diaphragm sniff ultrasound: Normal values, relationship with sniff nasal pressure and accuracy for predicting respiratory involvement in patients with neuromuscular disorders

**DOI:** 10.1371/journal.pone.0214288

**Published:** 2019-04-24

**Authors:** Abdallah Fayssoil, Lee S. Nguyen, Adam Ogna, Tanya Stojkovic, Paris Meng, Dominique Mompoint, Robert Carlier, Helene Prigent, Bernard Clair, Anthony Behin, Pascal Laforet, Guillaume Bassez, Pascal Crenn, David Orlikowski, Djillali Annane, Bruno Eymard, Frederic Lofaso

**Affiliations:** 1 Service de Réanimation médicale et unité de ventilation à domicile, CHU Raymond Poincaré, APHP, Université de Versailles Saint Quentin en Yvelines, Garches, France; 2 Institut de Myologie, AP-HP, centre de référence des maladies neuromusculaires Nord/Est/Ile-de-France, G-H Pitié Salpétriêre, Paris, France; 3 Center of Clinical Investigation Paris-Est, Pitié Salpetrière, APHP, ICAN, Sorbonne Université, Paris, France; 4 Service de Radiologie, CHU Raymond Poincaré, APHP, Université de Versailles Saint Quentin en Yvelines, Garches, France; 5 Service de Physiologie—Explorations fonctionnelles, CHU Raymond Poincaré, APHP, Université de Versailles saint Quentin en Yvelines, Garches, France; 6 Service de Neurologie, CHU Raymond Poincaré, APHP, Université de Versailles Saint Quentin en Yvelines, Garches, France; 7 Service de médecine aigue, CHU Raymond Poincaré, APHP, Université de Versailles Saint Quentin en Yvelines, Garches, France; 8 Centre d’Investigation clinique et Innovation technologique CIC 14.29, INSERM, Garches, France; University of Modena and Reggio Emilia, ITALY

## Abstract

**Background:**

In patients with neuromuscular disorders, assessment of respiratory function relies on forced vital capacity (FVC) measurements. Providing complementary respiratory outcomes may be useful for clinical trials. Diaphragm sniff ultrasound (US) is a noninvasive technique that can assess diaphragm function that may be affected in patients with neuromuscular disorders.

**Purpose:**

We aimed to provide normal values of sniff diaphragm ultrasound, to assess the relationship between sniff diaphragm US, vital capacity (VC) and sniff nasal pressure. Additionally, we aimed to evaluate the diagnostic accuracy of sniff diaphragm US for predicting restrictive pulmonary insufficiency.

**Materials and methods:**

We included patients with neuromuscular disorders that had been tested with a sniff diaphragm US and functional respiratory tests. Healthy subjects were also included to obtain normal diaphragm sniff ultrasound. We performed diaphragm tissue Doppler imaging (TDI) and time movement (TM) diaphragm echography combined with sniff maneuver.

**Results:**

A total of 89 patients with neuromuscular diseases and 27 healthy subjects were included in our study. In patients, the median age was 32 years [25; 50] and the median FVC was 34% of predicted [18; 55]. Sniff diaphragm motion using TM ultrasound was significantly associated with sniff nasal pressure, both for the right hemidiaphragm (r = 0.6 p <0.0001) and the left hemidiaphragm (r = 0.63 p = 0.0008). Right sniff peak TDI velocity was also significantly associated with FVC (r = 0.72, p<0.0001) and with sniff nasal pressure (r = 0.66 p<0.0001). Sniff diaphragm ultrasound using either TM mode or TDI displayed significant accuracy for predicting FVC<60% with an area under curve (AUC) reaching 0.93 (p<0.0001) for the right sniff diaphragm ultrasound in TM mode and 0.86 (p<0.001) for right peak diaphragm TDI velocity.

**Conclusion:**

Sniff diaphragm TM and TDI measures were significantly associated with sniff nasal pressure. Sniff diaphragm TM and TDI had a high level of accuracy to reveal respiratory involvement in patients with neuromuscular disorders. This technique is useful to assess and follow up diaphragm function in patients with neuromuscular disorders. It may be used as a respiratory outcome for clinical trials.

## Introduction

Respiratory and cardiac failures are the main causes of morbidity and mortality in patients with neuromuscular disorders. Assessment and monitoring of respiratory function relies on functional pulmonary tests. Forced vital capacity (FVC) is used as an indicator of global respiratory function. However, vital capacity (VC) is a late indicator of respiratory muscle involvement in neuromuscular disorders since VC may be normal until a significant decrease in respiratory muscle strength occurs [1]. Sniff nasal pressure and maximal inspiratory pressures, tests used to assess inspiratory muscle strength, may be affected despite a normal FVC [2, 3]. Providing complementary respiratory outcomes may be useful for clinical trials. Among the respiratory muscles, the diaphragm is the main inspiratory muscle. Its assessment classically relies on a trans-diaphragmatic pressure measurement. Since this test is invasive and not comfortable, it cannot be used routinely. Recent research has focused on ultrasound to noninvasively assess diaphragm muscle function [4, 5, 6]. Other techniques have been suggested to assess respiratory muscle function. These techniques include a ratio of maximal expiratory and inspiratory pressures (PEmax/PImax) [7] and the maximum relaxation rate (MRR) of inspiratory muscles [8]. Diaphragm function can be assessed using ultrasound at rest, during deep inspiration or during a sniff maneuver [9]. However, this technique is limited in the assessment of the left diaphragm during deep inspiration, due to loss of the ultrasound picture of the left diaphragm during deep inspiration. Sniff is a physiological maneuver that is used during fluoroscopy for the assessment of diaphragm function [10], it can be used to investigate motion of the right and left diaphragm, and it can be coupled with ultrasound. Additionally, in cardiology, tissue Doppler imaging (TDI) is used to assess subclinical myocardial impairment [11, 12]. Diaphragm sniff ultrasound may be affected earlier in patients with neuromuscular disorders. Little is known about the potential application of this technique in diaphragm muscle analysis. A subset of patients with muscular dystrophy may have subclinical diaphragm impairment with normal VC values.

In this study, we aimed to complete the following:

- To provide normal values for diaphragm peak sniff velocity using TDI and sniff diaphragm motion using time movement (M mode) ultrasound.
- To evaluate the relationship between diaphragm sniff ultrasound and sniff nasal pressure, as well as the relationship between sniff ultrasound and FVC.
- To determine whether peak TDI velocities may be reduced in neuromuscular patients with normal FVC.
- To assess the diagnostic accuracy of diaphragm sniff TDI and sniff M mode ultrasound for predicting impairment of respiratory muscles among patients with neuromuscular disorders.

## Materials and methods

We retrospectively included patients with neuromuscular disorders followed at Raymond Poincare hospital, a tertiary neuromuscular reference center specialized in the cardiac and pulmonary management of patients with neuromuscular disorders. We included patients who benefited from a sniff diaphragm ultrasound (right and/or left) and we recorded the functional respiratory tests available that included sniff nasal pressure and/or FVC. Diaphragmatic ultrasound was performed in patients with echocardiography, by the same operator (AF).

We recorded the following parameters from the medical records: age, sex, body mass index (BMI), type of disease, peripheral skeletal muscle insufficiency (Walton score), cardiac drugs, steroids, left ventricular ejection fraction (LVEF), history of documented sleep apnea (from polygraphy or polysomnography), functional lung tests that included forced FVC, decubitus FVC, forced expiratory volume in one second (FEV1), inspiratory capacity (IC), expiratory residual volume (ERV), maximal inspiratory pressure (MIP), maximal expiratory pressure (MEP), sniff nasal pressure and arterial partial carbon dioxide pressure (PaCO2). The study was performed in compliance with the ethical principles formulated in the Declaration of Helsinki and was approved by the *French National Agency regulating Data Protection (commission nationale de l'informatique et des libertés)*. All data were fully anonymized before analysis. We also included a healthy control group that underwent diaphragm ultrasound imaging, to obtain reference diaphragm ultrasound sniff values.

### Lung function tests

Pulmonary function testing was performed in all patients as a part of the routine evaluation, including spirometry that was performed according to ATS/ERS recommendations[13], using a Vmax 229 Sensormedics System (Yorba Linda, CA, USA) with the patient in the upright position [14]. Measurements are expressed as percent of predicted values [15]. Pulmonary function and pressure measurements were performed by different experimental technicians who worked in the same respiratory muscle laboratory.

### Sniff nasal pressure and maximal inspiratory pressure and expiratory pressure measurements

Maximal inspiratory pressure (MIP) and sniff nasal pressure (SNIP) were both measured from functional residual capacity (FRC) in a standard manner (sitting position) and maximal expiratory pressure (MEP) measured from total lung capacity, according to previously described methods. MIP is an isometric maneuver, while SNIP is a quasi-isometric maneuver [3, 16]. MIP was measured with a flanged mouthpiece with the maneuvers repeated at least four times or until two identical readings were obtained [17]. SNIP was measured during at least 10 and up to a maximum of 20 sniffs in a standard manner according to previously described methods [3]. Briefly, the plug used to obstruct the nostril was an eartip usually used for auditory evoked potentials (Eartips, 13 mm, Nicolet, Madison, WI). This plug was connected to a pressure transducer via a catheter (see below). The length of this catheter was reduced to the minimum length possible. Air leak was detected by obstructing the other nostril during an inspiratory maneuver and when present it was eliminated by adding waxed earplug material. Detailed instruction on how to perform the sniff maneuver was not necessary and may be counterproductive [18]. Patients were vigorously coached during the test maneuvers.

All pressure signals were measured using a differential pressure transducer (Validyne, Northridge, CA), amplified by a carrier amplifier (Validyne), and passed through an analog-digital board to a computer running Acqknowledge software (Biopac System, Santa Barbara, CA) which allows visual feedback to improve the sniff efficiency. The signal was digitized at 100 Hz. Subjects received strong verbal encouragement with visual feedback, as previous studies have suggested [19]. Values of MIP, MEP and SNIP were expressed in cmH_2_O.

### Diaphragm ultrasound technique

**Fig 1:**
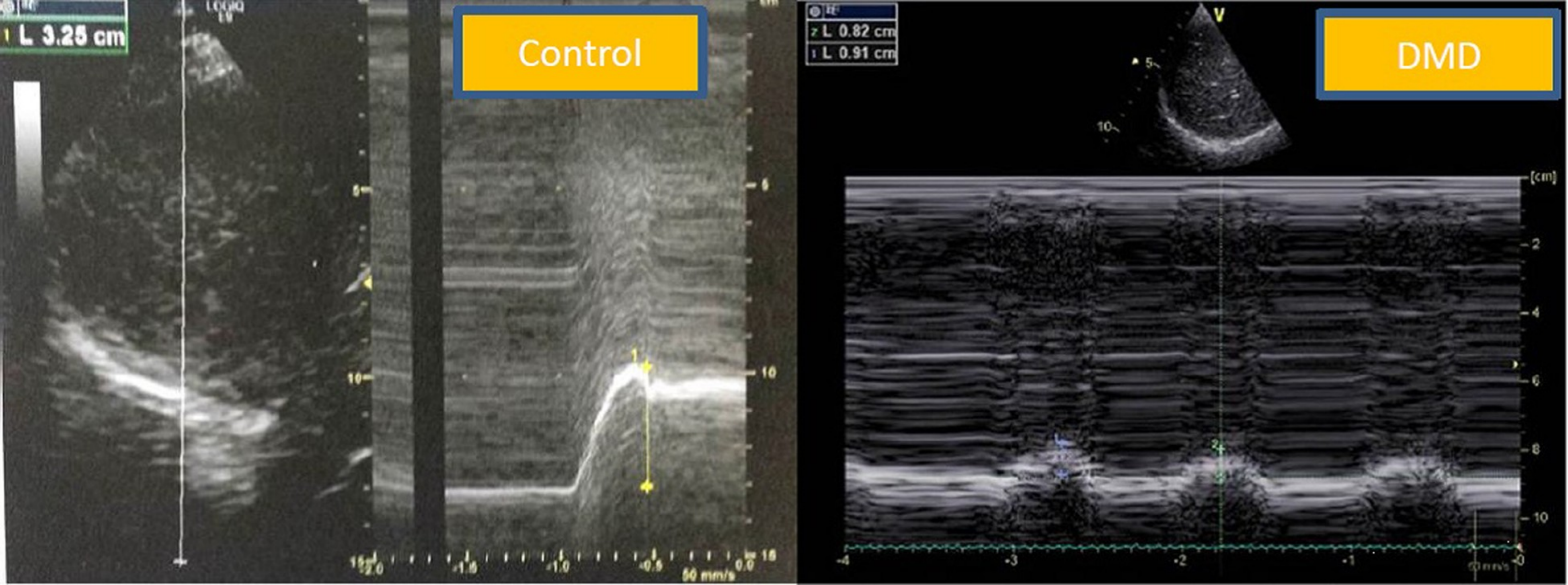
Diaphragm ultrasound displacement from the subcostal view in a control subject and in a DMD patient. Normal right diaphragm sniff time movement (TM) motion in the control group (*left)*; note the sharp upstroke during a sniff maneuver. Pathological right diaphragm motion during a sniff maneuver in a DMD patient (*right*). Note the reduced diaphragm motion with the sniff maneuver. DMD: Duchenne muscular dystrophy.

**Fig 2:**
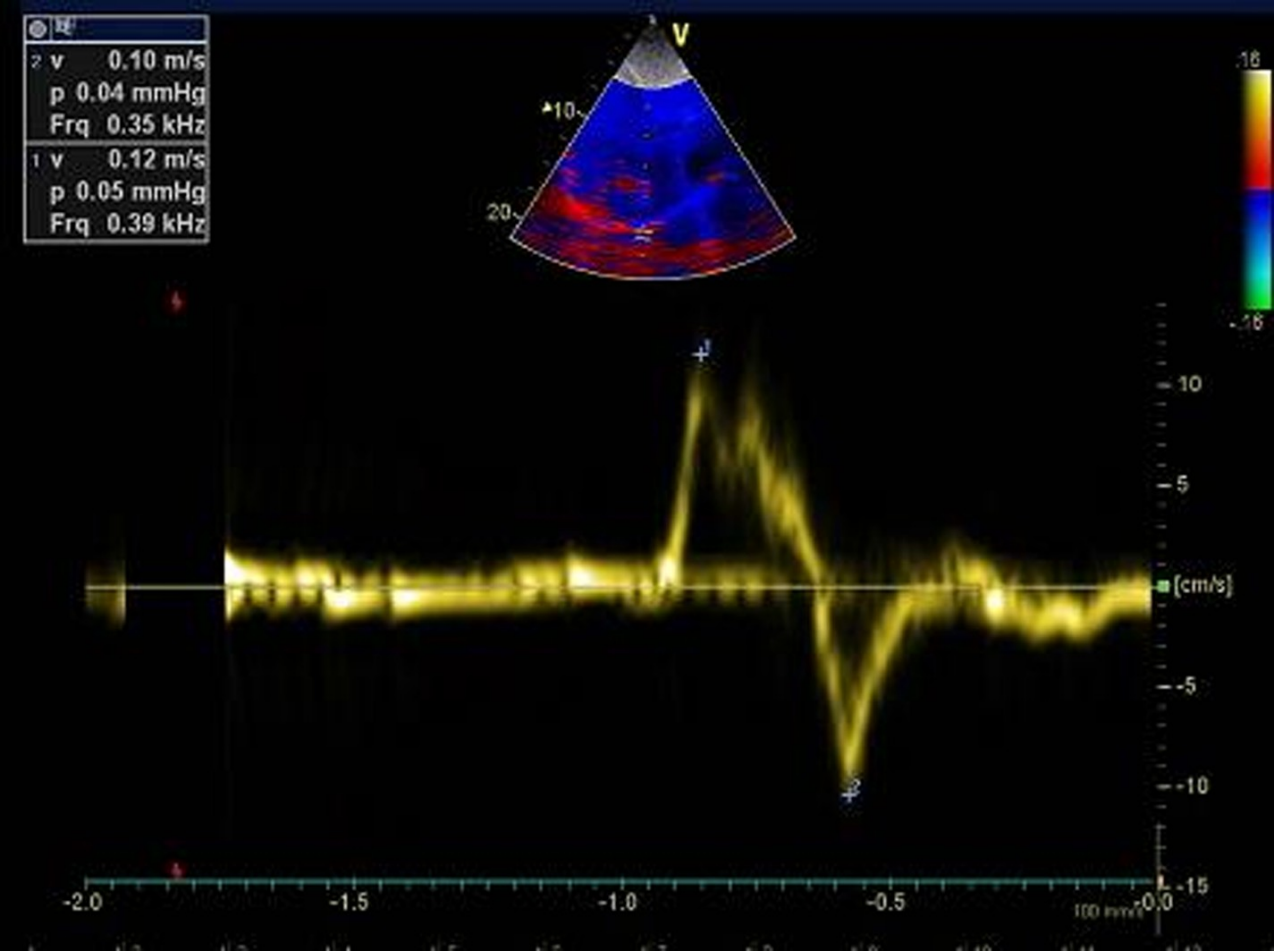
Sniff right hemidiaphragm tissue Doppler imaging peak velocity (arrow) in a volunteer (peak velocity = 12 cm/s).

**Fig 3:**
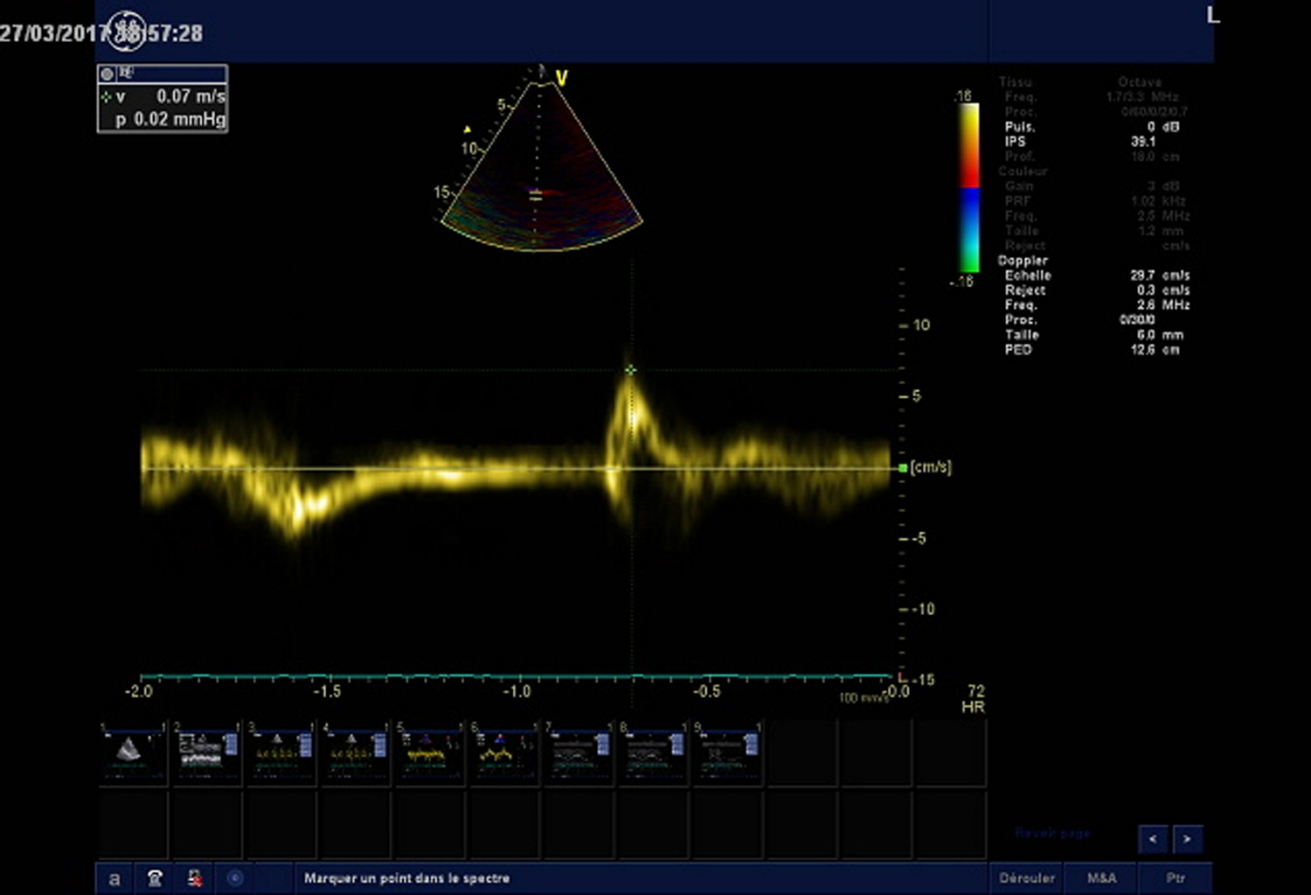
Pathological right hemidiaphragm peak tissue Doppler imaging velocity during a sniff maneuver in a DMD patient. Note the reduced peak sniff diaphragm TDI velocity (arrow).

Diaphragm ultrasound was performed in the supine position by the same operator (AF), who was blinded to the pulmonary function tests. We used the liver window for the right hemidiaphragm analysis and the spleen window for the left hemidiaphragm analysis. The transducer was placed in the anterior subcostal region between the midclavicular and anterior axillary lines so that the ultrasound beam reached the posterior part of the diaphragm. After visualization of the diaphragm, an M mode was applied perpendicularly to the hemidiaphragm to measure diaphragm motion during inspiration and during a sniff maneuver. Patients were in a semirecumbent position (45°) during the procedure. In normal subjects, the normal inspiratory motion is caudal and the operator recorded the M mode trace moving upward (diaphragm moving toward the probe). With the sniff maneuver, the operator recorded a sharp upstroke in the normal situation due to the high speed of diaphragm movement (**Fig 1**). For sniff peak diaphragm TDI recording, we used the liver window or the spleen window. Using a cardiac probe, we activated the tissue Doppler imaging modality with the beam positioned perpendicular to the diaphragm motion; then, we recorded the peak sniff inspiratory velocity (cm/s) (**Figs 2 and 3**). Diaphragm thickness was also recorded from the apposition zone ultrasound, using a linear probe positioned in the midaxillary line perpendicular to the chest wall. Thickness was measured at the end of a quiet expiration.

### Statistical analysis

Continuous variables are reported as the median ± interquartile range [IQR] and compared using nonparametric tests due to their distribution; categorical variables are described by number of subjects and percentage and compared by Fisher’s exact test. Spearman correlations were performed to evaluate associations between continuous variables. Discrimination performances of sniff M mode and sniff TDI regarding FVC<60% and FVC<30% were assessed using receiver-operator characteristic (ROC) analyses, and corresponding ROC curves were drawn. C-statistics corresponded to the areas under corresponding ROC curves. Figures were created and statistical analyses were performed using GraphPad Prism (GraphPad Software, Inc., State of California, USA).

## Results

### Clinical and respiratory functional tests and diaphragm ultrasound

A total of 89 patients with genetically confirmed neuromuscular diseases were included in our study (35% Duchenne muscular dystrophy (DMD), 29% myotonic dystrophy type 1, 12% sarcoglycanopathies). The median age was 32 years [25; 50] and the median BMI was 22 kg/m^2^. In this sample, 57% of patients were wheelchair bound. Patients disclosed a restrictive respiratory insufficiency pattern with a median FVC of 34% of predicted [18; 55]. In total, 63% of patients were on home mechanical ventilation (HMV). Inspiratory muscles were affected as attested by a median inspiratory capacity (IC) of 59% of predicted [16; 74], a median MIP of 22 cmH20 [14; 32] and a median sniff nasal pressure of 22 cmH20 [14;32]. Diaphragm ultrasound parameters were significantly reduced in patients. **Table 1**summarizes the clinical and respiratory data in the patients. **Table 2**summarizes the diaphragm ultrasound data in the patients vs the control group.

**Table 1:** Clinical and respiratory data in patients.

Parameters	Number *(%) o*r median *[IQR]*
Neuromuscular Diseases (N = 89)	
DMD	31 (35%)
DM1	26 (29%)
Sarcoglycanopathy	11 (12%)
Mitochondrial disease	4 (4.5%)
Congenital myopathy	4 (4.5%)
Spinal muscular atrophy	3 (3%)
Pompe disease	2 (2%)
Desminopathy	1 (1%)
Other diseases	7 (8%)
Age (y)	32 [25;50]
Wheelchair bound	51 (57%)
Walton score	6 [3;7]
BMI (kg/m^2^)	22 [14; 27]
Sleep apnea (N, %)	24 (27%)
FVC (%)	34 [18;55]
Decubitus FVC (%)	30 [18;47]
IC (%)	59 [16;74]
FEV1 (ml)	1280 [530;1765]
FEV1/VC	91 [81;97]
PEF (l/s)	3 [2;4]
ERV (ml)	420 [110;640]
PaCO2 (mmHg)	46 [42;50]
MIP (cmH20)	22 [14;32]
MEP (cmH20)	22 [15;36]
Sniff nasal pressure (cmH20)	22 [14;32]
HMV	56 (63%)

Data are presented as number (percentage) or median [IQR]

BMI: body mass index; IC: inspiratory capacity (%); PEF: peak expiratory flow; FEV1: forced expiratory volume in one second; MIP: maximal inspiratory pressure; MEP: maximal expiratory pressure; PaCO2: arterial partial carbon dioxide pressure (mmHg); FVC: forced vital capacity (%); HMV: home mechanical ventilation; DMD: Duchenne muscular dystrophy; DM1: myotonic dystrophy type I. Y = years.

**Table 2:** Diaphragm ultrasound in the patient and control groups.

Ultrasound parameters	Patient group (N = 89)	Control group (N = 27)	p
Sex (F)	11 (12%)	14 (50%)	0.14
Age (y)	32 [25;50]	31 [26;39]	0.43
BMI (kg/m^2^)	22 [14;27]	22 [20;25]	0.46
Right deep inspiratory motion (mm)	23 [10;38]	72 [62;88]	<0.001
Left deep inspiratory motion (mm)	23 [11;36]	62 [61;97]	0.002
Right sniff TM diaphragm motion (mm)	12 [6;19]	32 [19;36]	<0.001
Left sniff TM diaphragm motion (mm)	13 [9;18]	33 [23;42]	0.001
Peak right sniff TDI diaphragm velocity (cm/s)	6 [4;9]	13 [10;14]	<0.001
Peak left sniff TDI diaphragm velocity (cm/s)	7 [5.5;11]	12 [11;13]	0.06
Right diaphragm thickness (mm)*	1.8 [1.5;2.1]	1.5 [1.3;1.9]	0.11
Left diaphragm thickness (mm)*	1.6 [1.4;2]	1.5 [1.1;1.9]	0.25

Data are presented as number (percentage) or median [IQR]

F: female; BMI: body mass index; TM: time movement; TDI: tissue Doppler imaging.

*: at rest. p = p value.

### Relationship between sniff ultrasound and sniff nasal pressure

**Fig 4:**
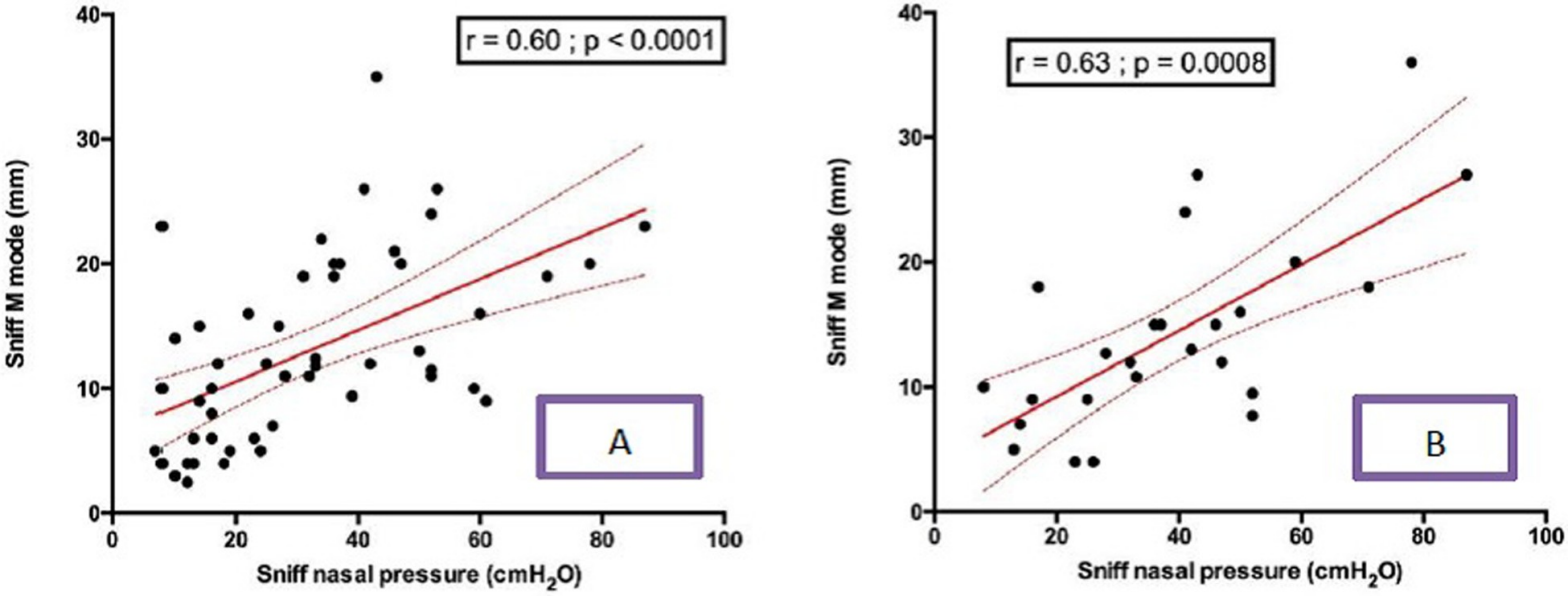
**Relationship between right diaphragm sniff TM motion and sniff nasal pressure in patients** (A), and the **relationship between left diaphragm sniff TM motion and sniff nasal pressure in patients** (B). TM = time movement; Sniff TM mode = Sniff TM motion.

**Fig 5:**
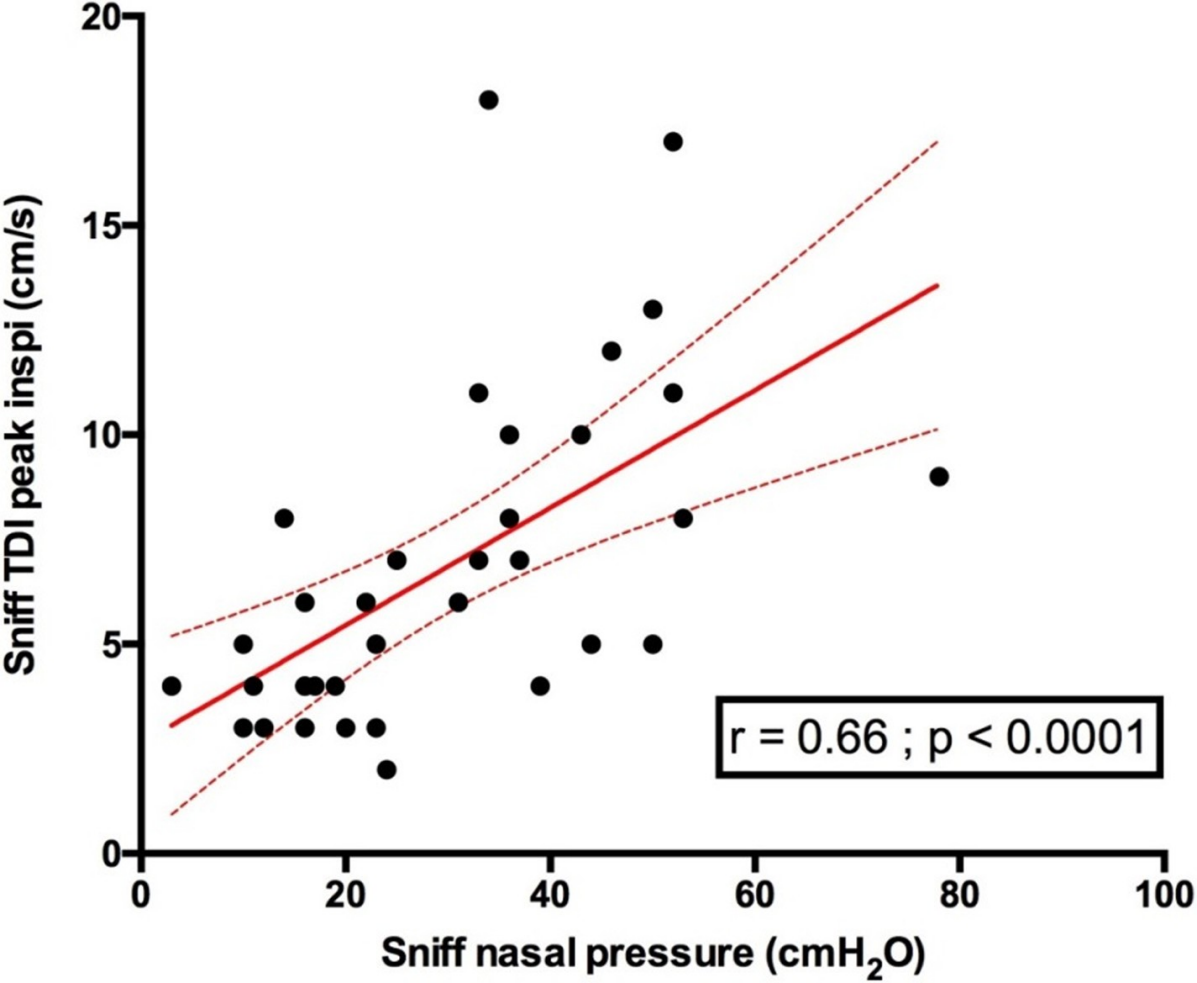
Relationship between right diaphragm sniff peak TDI velocity and sniff nasal pressure in patients. Sniff TDI peak inspi = sniff peak diaphragm tissue Doppler imaging velocity.

Diaphragm sniff time movement (TM) ultrasound motion was significantly associated with sniff nasal pressure, both for the right hemidiaphragm (r = 0.6 p <0.0001) and the left hemidiaphragm (r = 0.63 p = 0.0008) (**Fig 4**). Indeed, diaphragm peak sniff TDI velocities were significantly associated with sniff nasal pressure (**Fig 5**).

### Relationship between diaphragm sniff ultrasound and forced vital capacity

**Fig 6:**
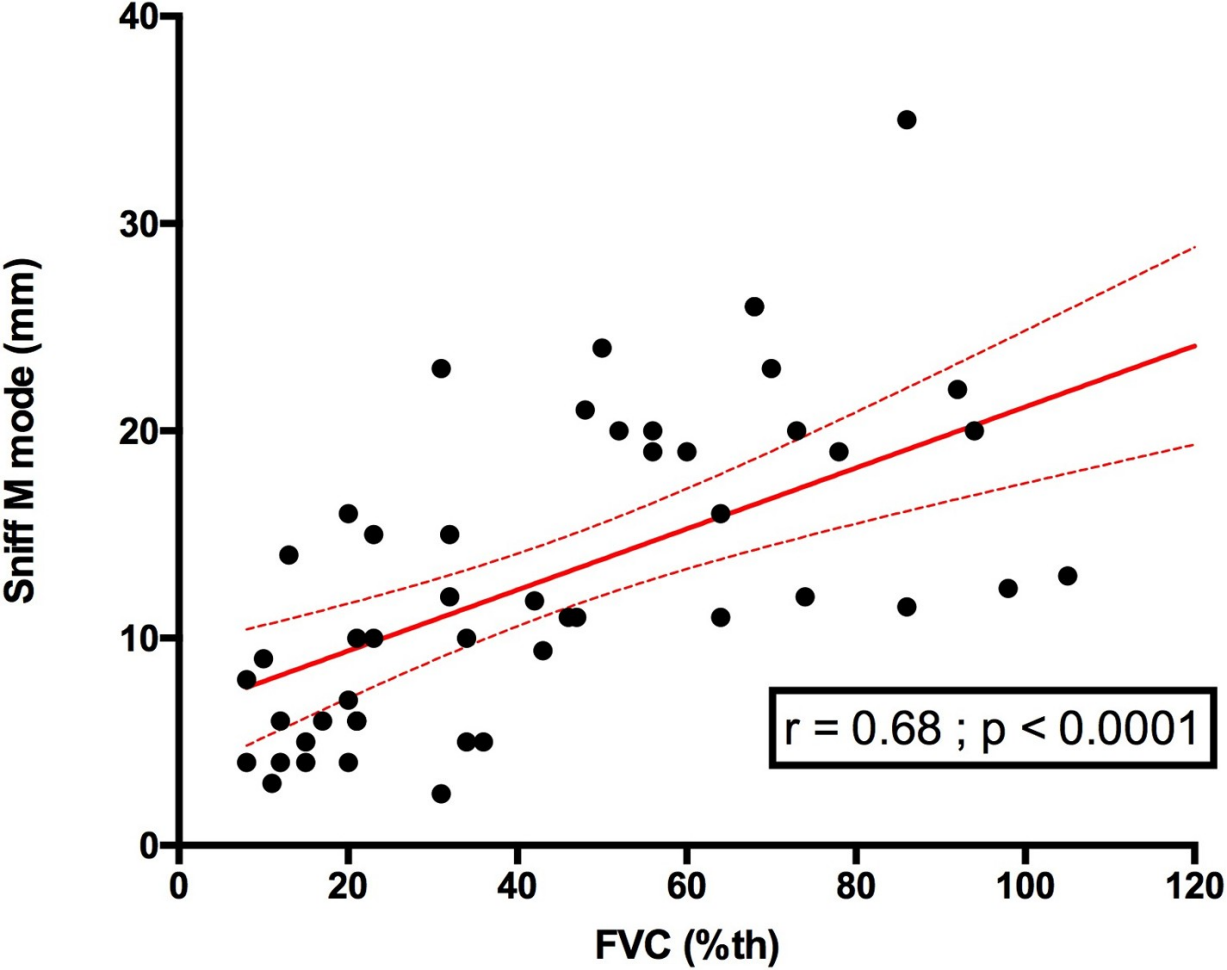
Relationship between right sniff TM mode and FVC in patients. FVC: forced vital capacity (%).

**Fig 7:**
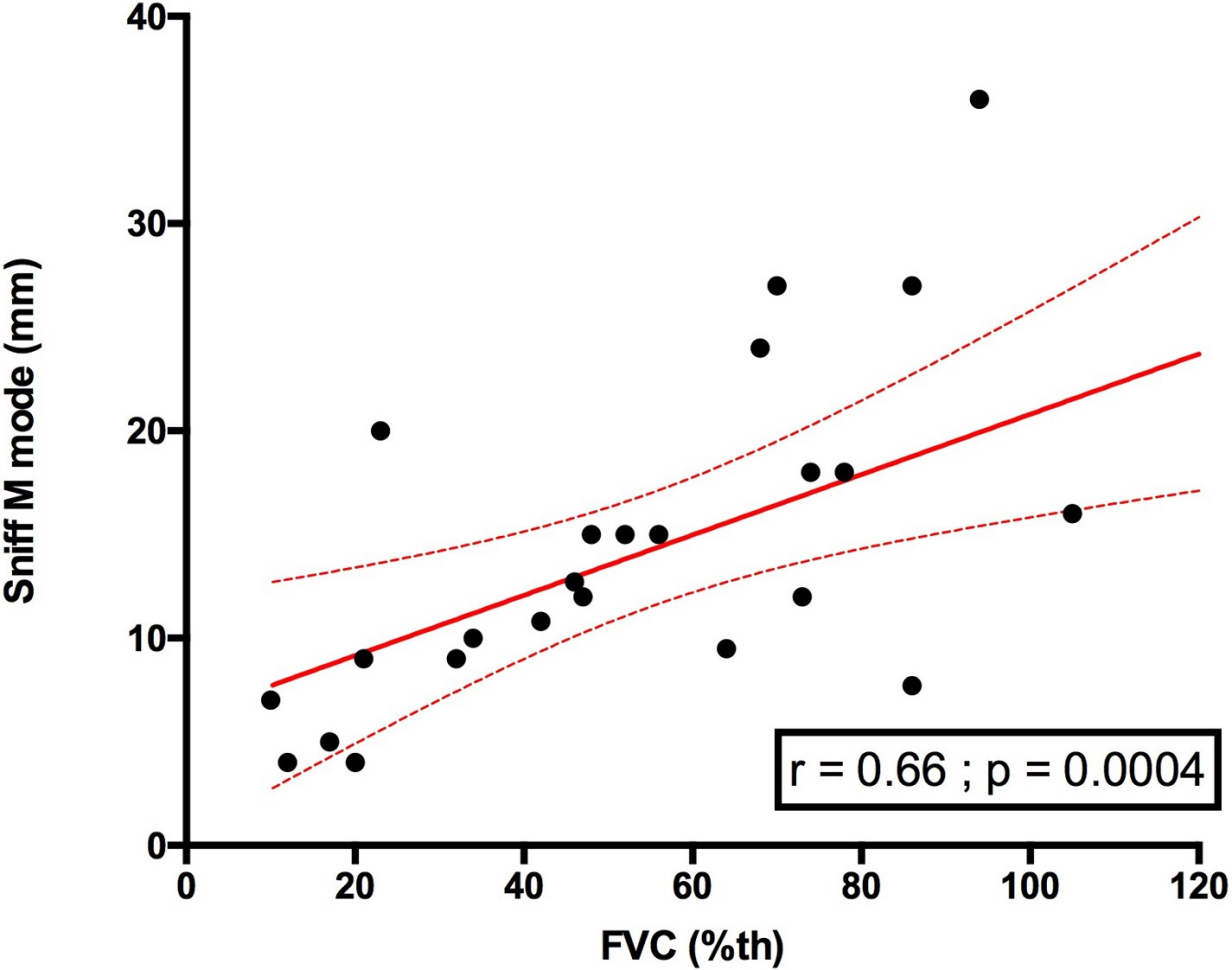
Relationship between left sniff TM mode and FVC in patients. FVC: forced vital capacity (%).

**Fig 8:**
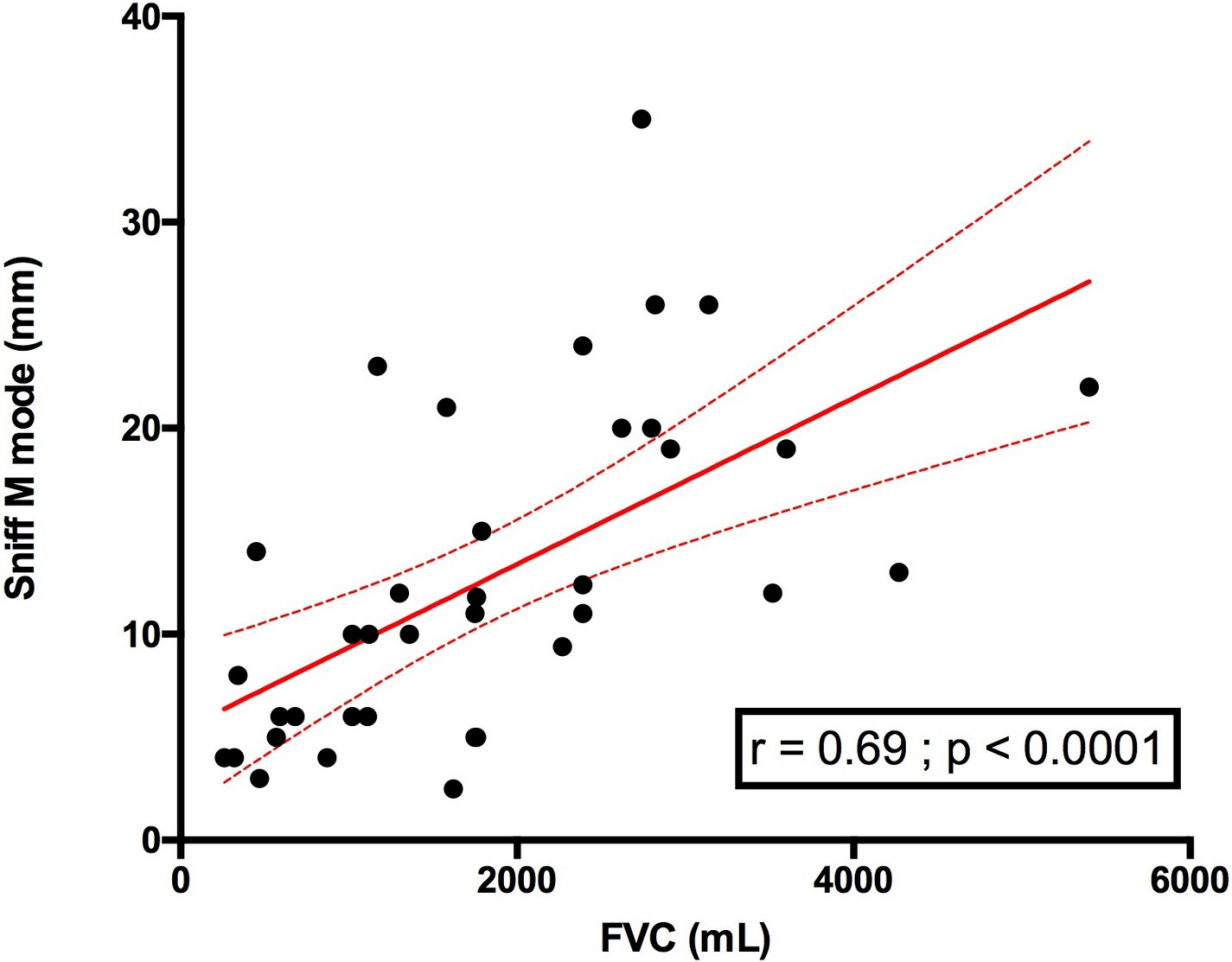
Relationship between right sniff TM mode and FVC (ml) in patients. FVC: forced vital capacity.

Using the global respiratory function assessment, diaphragm sniff ultrasound TM motion was significantly associated with FVC. This finding involved the right hemidiaphragm as well as the left hemidiaphragm (**Figs 6 and 7**). We also compared sniff M mode with FVC expressed in ml and observed a similar relationship to FVC expressed in %, which takes into account height (**Fig 8**).

**Fig 9:**
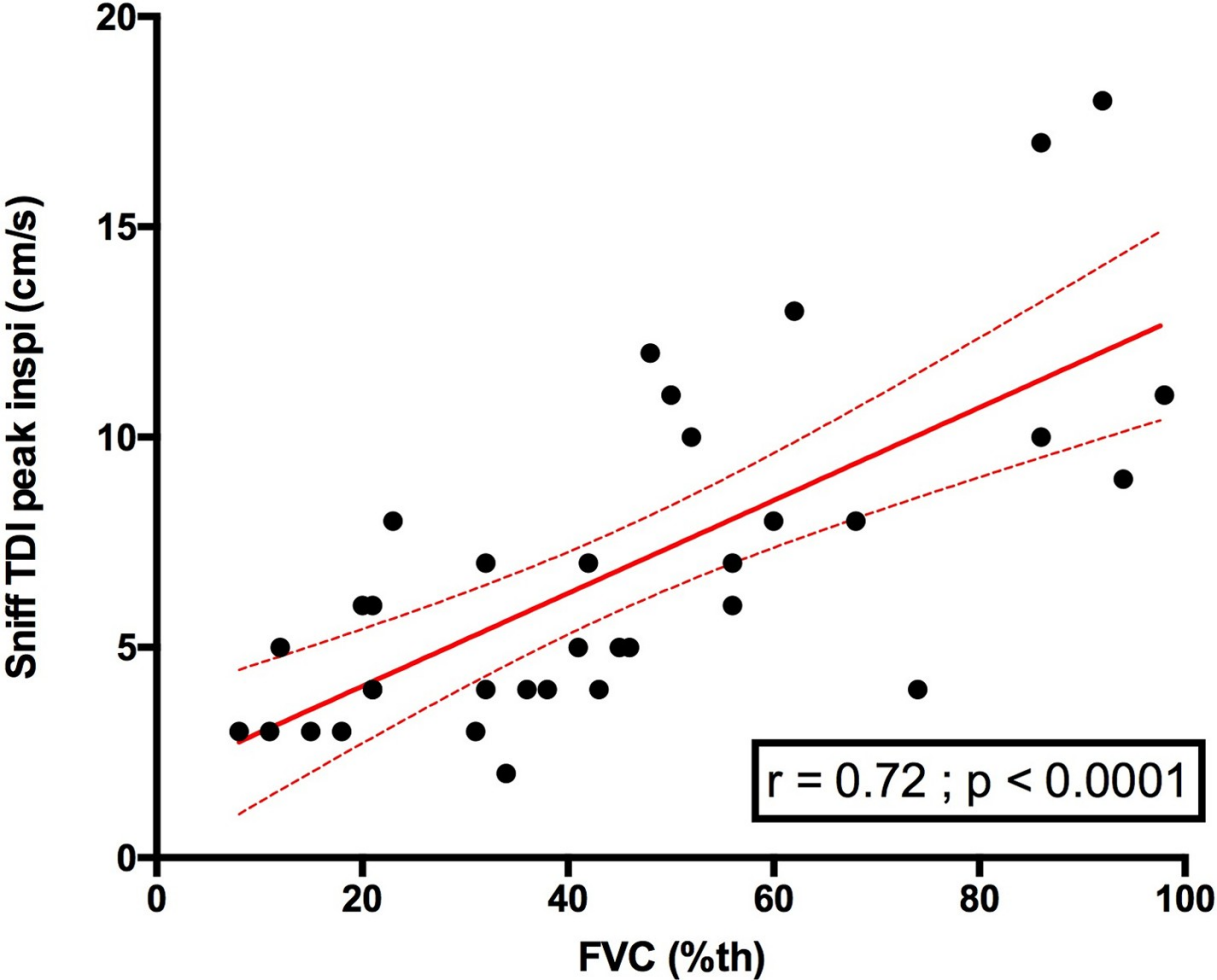
Relationship between right sniff TDI peak velocity (cm/s) and FVC in patients. FVC = forced vital capacity (%).

**Fig 10:**
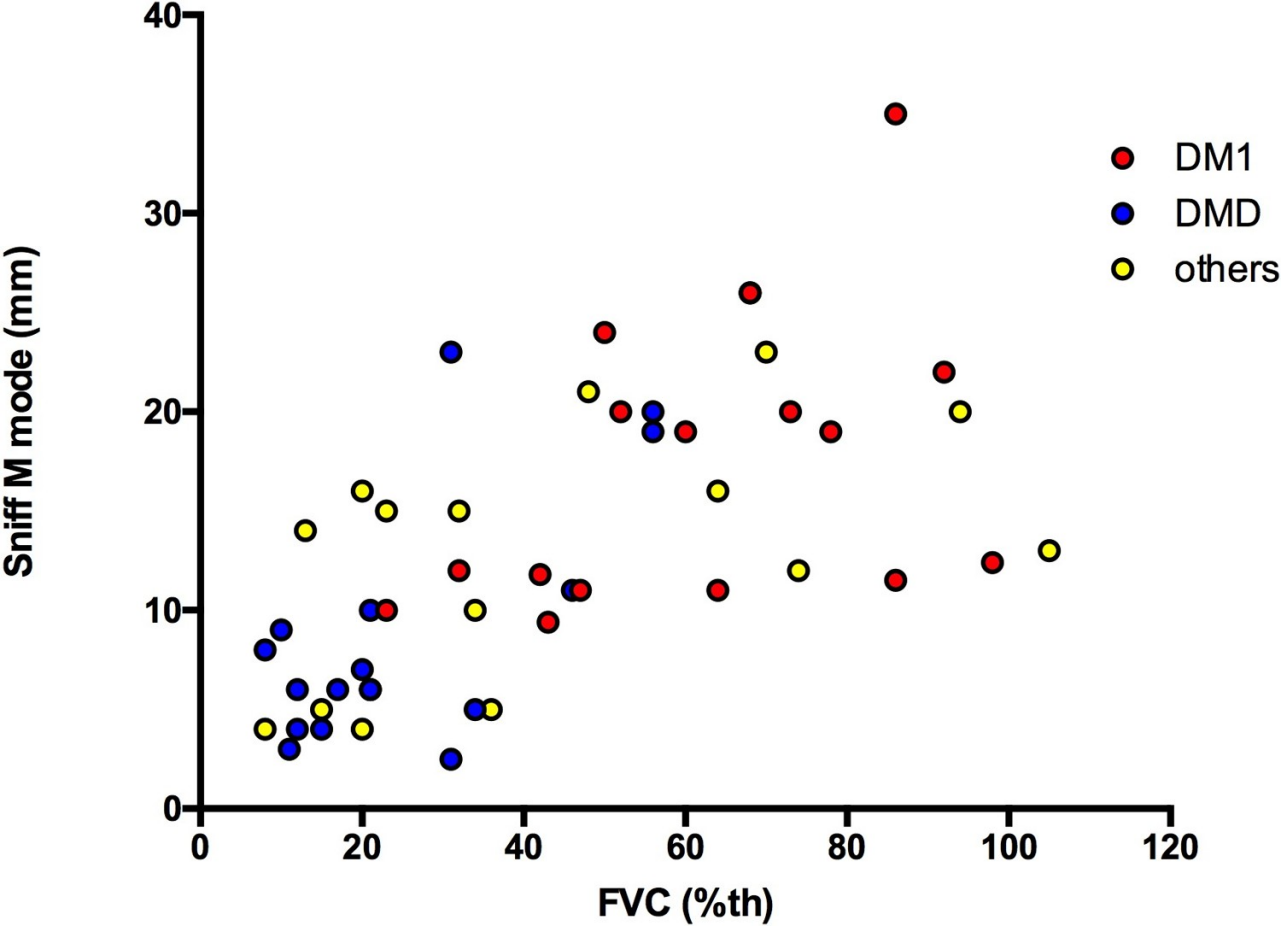
Relationship between right sniff diaphragm TM motion and FVC in DMD. **DM1 and other myopathies.** DMD = Duchenne muscular dystrophy; DM1 = myotonic dystrophy type I; others = other myopathies; FVC = forced vital capacity (%); sniff M mode = right sniff diaphragm time movement motion (mm).

Right sniff peak TDI was also significantly associated with FVC (r = 0.72, p<0.0001) (**Fig 9**). Supine FVC was also significantly associated with sniff diaphragm right TM motion (r = 0.59, p = 0.0003, n = 34). Additionally, Supine FVC was significantly associated with right diaphragm sniff TDI velocity (r = 0.59, p = 0.0007, n = 29). The level of the relationship between the sniff diaphragm TM motion and FVC remained similar in patients with DMD vs patients with DM1 vs patients with other myopathies (**Fig 10**).

### Sniff diaphragm TDI velocities and TM motion in patients with FVC>80%

**Figs 6, 7 and 9**provide an overview of the distribution of the right sniff TM motion and the right sniff peak TDI velocity in patients with FVC>80%. Among those with FVC>80%, 5 (83.3%) patients had a right diaphragm sniff TM motion under the median value obtained in controls. In patients with FVC>80%, 3 (60%) patients a had right sniff TDI peak velocity under the median right diaphragm sniff TDI peak velocity obtained in the control group.

### Accuracy of sniff ultrasound for the prediction of respiratory muscle impairment in patients with muscular dystrophy

Using receiver operating characteristic (ROC) curves, sniff diaphragm ultrasound using either TM mode or TDI accurately discriminated those with FVC<60%; the area under the curve (AUC) reached 0.93 (p<0.0001) for the sniff right TM mode diaphragm ultrasound and 0.86 (p<0.001) for right peak diaphragm TDI velocity. In patients with significant respiratory insufficiency (FVC<30%), the AUC remained high but with a slight decrease in the AUC of the right diaphragm ultrasound TDI (0.76, p<0.017). The M mode right diaphragm motion value cutoff to predict a FVC<60% was 25 mm with a sensitivity of 100% and a specificity of 64%. The M mode right diaphragm motion value cutoff to predict a FVC<30% was 10.5 mm with a sensitivity of 84% and a specificity of 84%.

**Fig 11:**
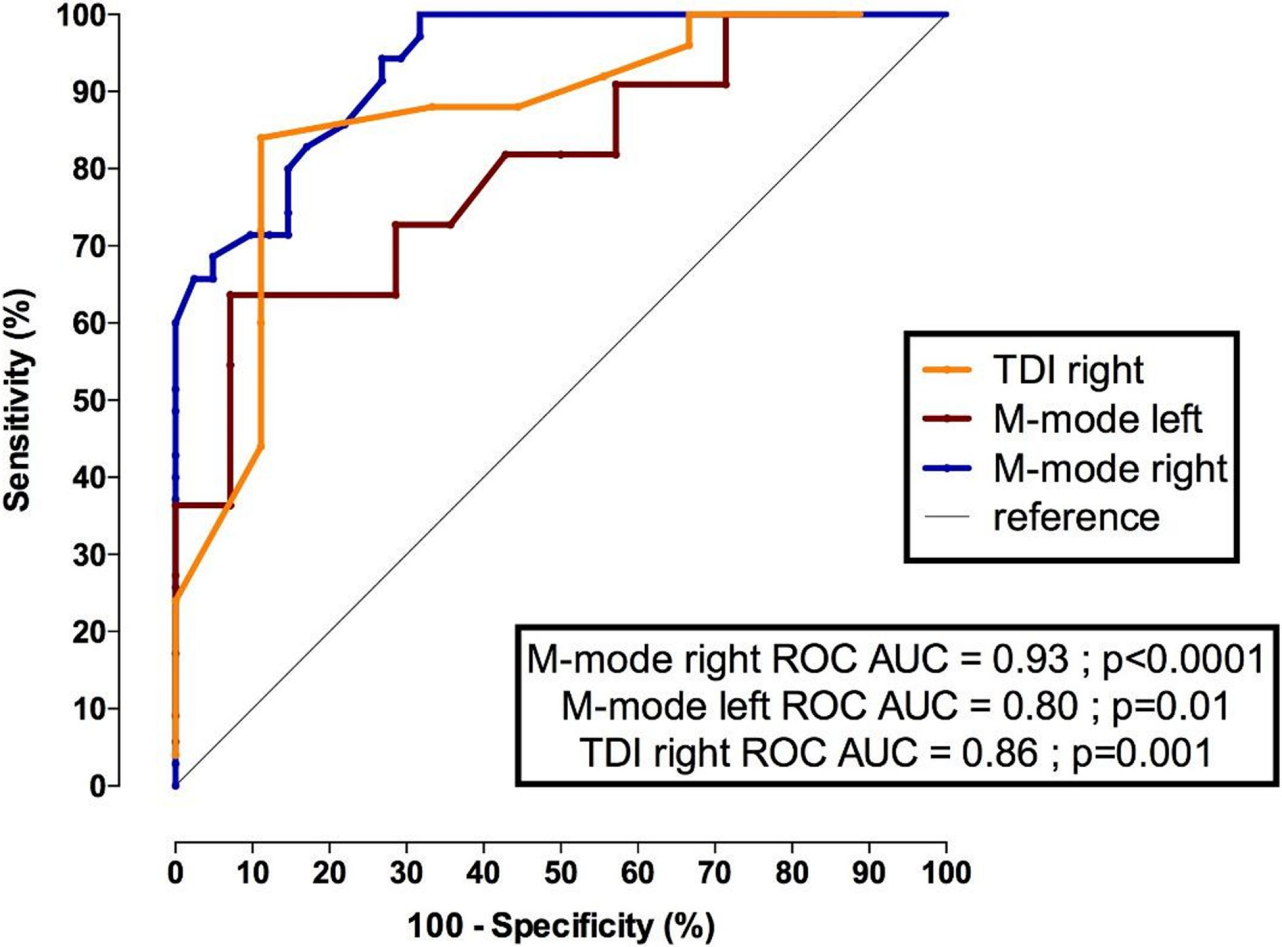
ROC (receiver operating characteristic) curves for predicting a FVC<60% in patients with neuromuscular disorders. TDI right: peak sniff TDI velocity (cm/s) at the right hemidiaphragm; M mode left: left diaphragm motion during a sniff maneuver (mm); M mode right: right diaphragm motion during a sniff maneuver (mm); FVC = forced vital capacity (%).

**Fig 12:**
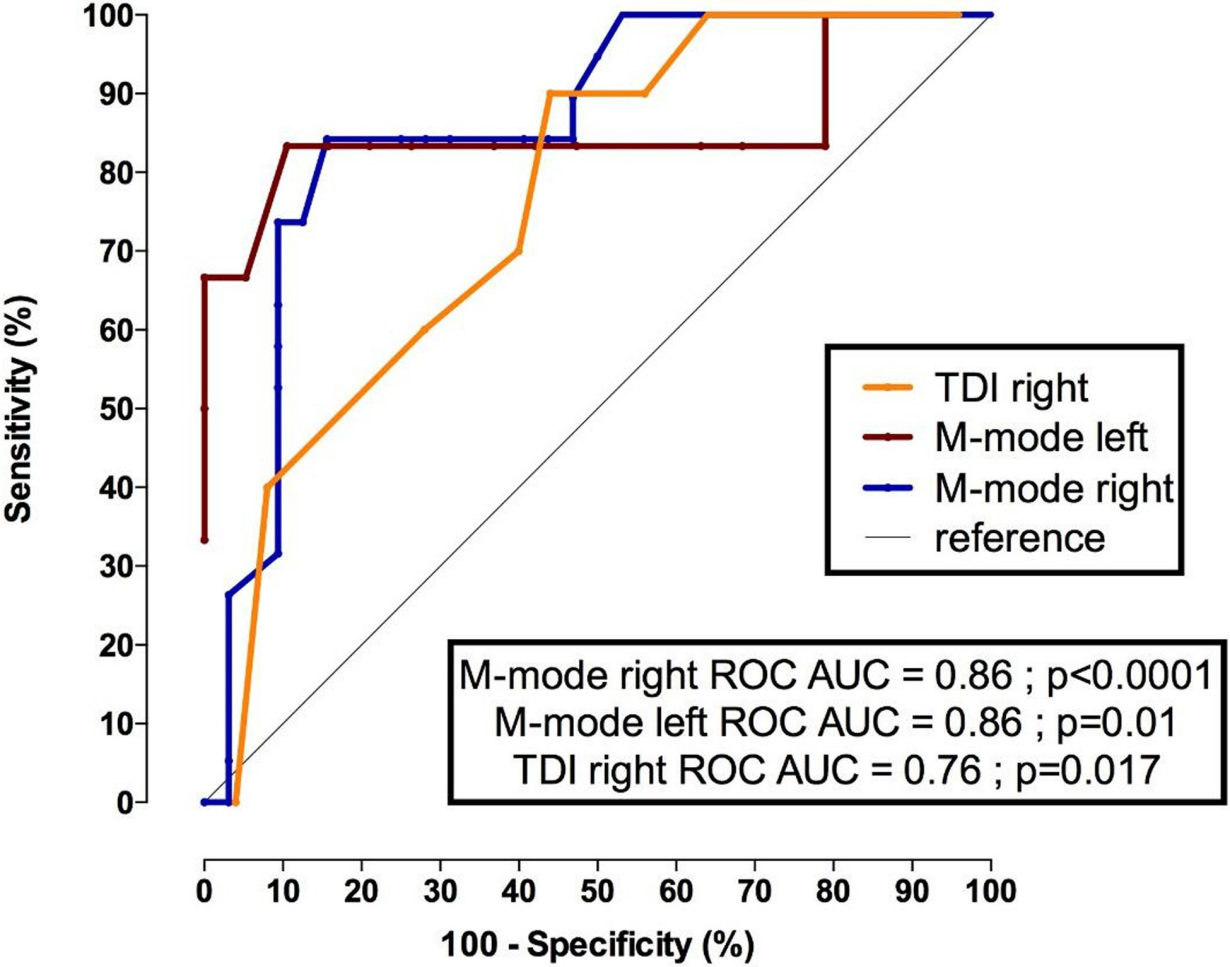
ROC curves for predicting a FVC <30% in patients with neuromuscular disorders. M mode right: right diaphragm motion during a sniff maneuver; M mode left: left diaphragm motion during sniff maneuver (mm); TDI right: peak sniff TDI velocity (cm/s) recorded at the right hemidiaphragm; FVC: forced vital capacity (%).

The right peak TDI velocity value cutoff to predict a FVC<60% was 7.5 cm/s with a sensitivity of 84% and a specificity of 89%. The right peak TDI velocity value cutoff to predict a FVC<30% was 6.5 cm/s with a sensitivity of 90% and a specificity of 56%. **Figs 11 and 12**show the ROC curves of the sniff diaphragm ultrasound for predicting FVC<60% and for predicting FVC<30%, respectively.

## Discussion

In the present study, we describe for the first time the diaphragmatic sniff ultrasound pattern in patients with muscular dystrophy, investigating the relationship between sniff diaphragm ultrasound and sniff nasal pressure as well as the relationship with FVC. We also report the accuracy of sniff diaphragm ultrasound for predicting moderate and/or severe respiratory muscle impairment. The main findings are the following:

- Sniff diaphragm ultrasound motion using TM mode was significantly reduced in patients with myopathies and was correlated with FVC and sniff nasal pressure.
- Sniff peak diaphragm ultrasound TDI velocity was significantly reduced in patients with myopathies and was correlated with FVC and sniff nasal pressure.
- Diaphragm peak sniff TDI velocities and diaphragm sniff TM motion may be impaired in patients even with FVC>80%.

Lung volumes in patients with neuromuscular disorders are affected by weakness of the respiratory muscles [20], and FVC provides a global evaluation of respiratory muscle function. In patients with NMD, the classic pattern of restriction is a reduction of FVC [21]. FRC may be normal or decreased, and RV may increase [22]. In our study, FVC was significantly associated with sniff diaphragm ultrasound. Interestingly, we found that sniff diaphragm ultrasound may predict restrictive impairment in patients with muscular dystrophy and was associated with sniff nasal pressures. Classically, MIP and sniff nasal pressures have been used to assess inspiratory muscle strength in respiratory laboratories [20]. Low values may be due to muscle failure, however, they may also be due to a lack of motivation, lack of coordination, peribuccal leaks and fatigue [23]. The sniff test is a natural effort maneuver that solves leak problems, reduces the risk of fatigue and can be combined with diaphragm ultrasound. However, the sniff value can be underestimated in cases of nasal obstruction [23]. Our data support the fact that sniff TDI can be used to detect early subclinical diaphragm involvement in patients with neuromuscular diseases. Indeed, the usefulness of TDI is to depict early muscle involvement in a pathological setting. Similar to the way TDI is used in cardiology to detect early myocardiopathy before the onset of a reduced ejection fraction [12], TDI may be used in pneumology and combined with sniff maneuver to detect early muscle involvement in myopathy before the onset of reduced FVC. In the early stages of diaphragm involvement, FVC remains normal, since FVC is a late marker of respiratory muscle involvement. In our study, in the control group, the median normal peak sniff TDI was 13 cm/s for the right hemidiaphragm and 12 cm/s for the left hemidiaphragm, and some patients had a reduced peak sniff TDI velocity, and at the same time, had a forced VC that was in the normal range. These findings are interesting since they could prompt monitoring in patients with abnormal sniff TDI. Patients with neuromuscular disorders presenting abnormal diaphragm peak TDI velocity may benefit from close pulmonary test monitoring. In normal subjects, during the sniff maneuver, inspiratory muscles are short at higher speed, and the pressure measured in the esophagus is closely related to the pressure in the mouth, nasopharynx and nose [24]. Heritier *et al*[3] reported an excellent correlation between sniff nasal pressure and sniff esophageal pressure (r 0.99, p<0.001). Diaphragm ultrasound emerged recently in the literature as a noninvasive method to assess diaphragm function [25, 26]. Our data in the control group regarding sniff TM diaphragm motion were similar, even higher than the data reported by Boussuges *et al* [27], 16 mm in women and 18 mm in men [27]. Various factors (sex and height) may influence normal diaphragm ultrasound, as reported by Scarlata *et al* [25]. In our study, we also compared sniff M mode with FVC expressed in ml and observed a similar relationship to FVC expressed in %, which takes into account height. No data have been reported regarding sniff diaphragm TDI velocities in the literature. In our study, the correlation between peak sniff TDI velocity and sniff nasal pressure was high (r = 0.66 p<0.0001) but was relatively reduced in comparison with the correlation between sniff esophageal pressure and sniff nasal pressure reported by Heritier *et al* [3](r = 0.99). This discrepancy may be explained by the fact that the peak sniff TDI velocity recorded the contribution of one side of the diaphragm and the peak velocity may be influenced by the pressure interactions between the chest and abdomen, the geometry and pattern of the ribcage. Moreover, the sniff maneuver used during ultrasound was different from the method used in pulmonary laboratories since the patient has two nostrils open during diaphragm sniff ultrasound examinations. Finally, the AUC for sniff diaphragm ultrasound using TDI or TM mode was significantly high for predicting FVC <60% and/or FVC<30%. However, in patients with FVC<30%, the sniff right TDI velocity displayed a lower AUC (0.76, p 0.017), in comparison with sniff right TM mode diaphragm displacement (AUC 0.86, p<0.0001). This difference can be explained by the lower TDI velocity recorded for the diaphragm with sniff, which sometimes rendered the recording curve analysis difficult.

### Clinical perspective

The assessment and monitoring of diaphragm function requires dedicated equipment and specialized techniques, which are not available in all centers. A single sniff diaphragm ultrasound measuring using TDI or TM mode can help physicians detect patients with asymptomatic restrictive pulmonary insufficiency and early diaphragm involvement in patients with neuromuscular disorders. Diaphragm sniff TDI may be used as a presymptomatic test to reveal early diaphragm involvement in patients with neuromuscular disorders. These findings may help clinicians select patients for regular respiratory test evaluation during follow-up, particularly in patients with TDI velocity impairment. Our study provided normal sniff diaphragm ultrasound values. These findings can be used as reference values for other studies in the neuromuscular field. Sniff diaphragm ultrasound can be used as a screening test and could be used as a monitoring tool for future biotherapy assessment. In patients with significant respiratory insufficiency, it would be appropriate to focus on the right TM mode technique for monitoring patients. Finally, studies that assess economic performance and nursing technical requirements need to be performed with this new technique. In the future, this radiological technique may be considered as an additional test in the arsenal of pulmonary exploratory functional tests used in a multiparametric approach in the neuromuscular field.

### Limits

This study is limited by its retrospective design. Additionally, sniff ultrasound is a volitional test and data depend on patient motivation and fatigue. The other limitation relies on the technical aspect of Doppler imaging. Indeed, velocity recorded with TDI depends on beam angulation and the beam direction should be perpendicular to diaphragm displacement to obtain the maximal TDI peak velocity. Moreover, patient position may affect respiratory function tests [28]. Here, with US, patients were in a semirecumbent position (45°) that would amplify diaphragm dysfunction and US measurements [27, 29]. The supine position is preferred, because there is less overall variability, less side-to-side variability, and greater reproducibility. Additionally, diaphragm excursion is known to be greater in the supine position for the same volume inspired than in the sitting or standing positions because the abdominal viscera more easily moves the diaphragm in this position, and the relationship between inspired volume and diaphragm movement has been shown to correlate better in the supine position than in the sitting position. The supine position also exaggerates any paradoxical movement and limits any compensatory active expiration by the anterior abdominal wall, which may mask paralysis [4]. According to Brown *et al* [29], intraobserver reliability was excellent (>0.93) for all body positions tested. Because many of our patients were not able to maintain the strict supine position for different reasons (e.g., tolerability), we systematically used the semirecumbent position (45°). Nevertheless, despite the difference in position between respiratory function measurements (90°) and diaphragmatic ultrasound measurements (45°), significant correlations were observed. Moreover, we compared nasal pressure and diaphragm velocity and motion. These latter measures depend on other factors that include abdominal pressure and compartment and history of abdominal surgery, and we did not have esophageal pressure for the reference method. Finally, nasal pressure does not necessarily reflect esophageal pressure in some patients with neuromuscular disease due to the inability to generate a significant transnasal pressure [30].

## Conclusion

Sniff diaphragm TM and TDI are significantly associated with sniff nasal pressure. Sniff diaphragm TM and TDI had high accuracy for depicting respiratory involvement in patients with neuromuscular disorders. This noninvasive diaphragm evaluation can reveal early diaphragm involvement. These techniques are useful to assess and follow up diaphragm function in patients with neuromuscular disorders and may be used as a respiratory outcome for clinical trials.
